# Evaluation of Subchronic Oral Dose Toxicity of Freeze-Dried Skimmed Powder of *Zophobas atratus* Larvae (frpfdZAL) in Rats

**DOI:** 10.3390/foods9080995

**Published:** 2020-07-24

**Authors:** Sun Young Kim, Kyu-Won Kwak, Eun-Sung Park, Hyung Joo Yoon, Yong-Soon Kim, Kwanho Park, Eunsun Kim, Sun-Don Kim

**Affiliations:** 1 Industrial Insect Division, Department of Agricultural Biology, National Institute of Agricultural Sciences, Rural Development Administration, Wanju 55365, Korea; carp0120@korea.kr (S.Y.K.); nawoon23@korea.kr (K.-W.K.); yoonhj1023@korea.kr (H.J.Y.); kaiko0214@korea.k (Y.-S.K.); nicegano@korea.kr (K.P.); ensuny88@korea.kr (E.K.); 2 Nonclinical Research Institute, Chemon Inc., 240, Nampyeong-ro, Yangji-myeon, Cheoin-gu, Yongin-si, Gyeonggi-do 17162, Korea; buffett21@chemon.co.kr

**Keywords:** edible insect, food safety, adverse effects, allergic reaction

## Abstract

*Zophobas atratus* (Coleoptera: *Tenebrionidae*), the giant mealworm beetle, is known as an edible insect containing a high protein content which may serve as new sources of human food and animal feed. However, potential toxicity and food safety analyses of *Z. atratus* have not been previously investigated. Therefore, in this study, we aimed to evaluate toxicity of freeze-dried skimmed powder of *Z. atratus* larvae (frpfdZAL), known as the super mealworm. Toxicological assessments were performed at the doses of 1250, 2500, and 5000 mg/kg/day in a 2- and a 13-week oral repeated-dose toxicity study of frpfdZAL in male and female Sprague-Dawley (SD) rats in accordance with the Organisation for Economic Co-operation and Development (OECD) guidelines and the principles of Good Laboratory Practice (GLP). No toxicological changes in clinical signs, body weights, water and food consumption, urinalysis, hematology, clinical biochemistry, gross findings, and histopathological examinations were observed. In conclusion, the no-observed-adverse-effect level (NOAEL) of frpfdZAL was 5000 mg/kg/day and target organ was not identified in both sexes of rats. In addition, frpfdZAL did not induce increases of serum ImmunoglobulinE (IgE), an identifier of allergic reactions in rats. Collectively, these results suggest that frpfdZAL is safe with no adverse effects, and able to be applied as an edible ingredient or other biological uses.

## 1. Introduction

The Food and Agricultural Organization of the United Nations (FAO) has been addressing topics related to edible insects since 2003 [1]. As a new source of protein or an alternative to animal proteins, insects have several advantages such as environmental sustainability, short life cycles, and ease of handling and growing [2,3,4]. Insect bodies contain high quality proteins, polyunsaturated fatty acids, minerals and vitamins, etc.; more than 1500 insect species are known to be consumed in at least 113 countries [5,6]. At present, in Europe and the United States, the list of industries seeking to use edible insects in the development of alternative foods and new drugs is growing rapidly [7]. There is also a new technology to develop new foods through the method of using processed insect proteins [8]. Despite the utilization of edible insects having many advantages, the absence of an overall system, such as standardization and quality control of edible insects and government regulations guaranteeing the safety and valid expiration period for edible insect-food is a major obstacle to the edible insect industry [9].

*Zophobas atratus* (*Z. atratus* (Coleoptera: *Tenebrionidae*)), the giant mealworm beetle, is imported to Korea as a source of protein for arthropods, birds, amphibians, and small mammals [10,11], and is well-known to contain a high protein content [12]. In addition, *Z. atratus* has been established as a sustainable source of animal protein source in human civilizations [13]. It has been reported that *Z. atratus* can be used as a high-protein content product in food and animal feed, with advantages being its high nutritional value and low price [14]. In the same Tenebrionidae family, *Tenebrio molitor* larvae are also considered suitable for human consumption [15,16,17]. An evaluation of the safety of *T. molitor* larvae as a food ingredient revealed no adverse effect or toxicity in Sprague–Dawley (SD) rats after 90 days of administration at a maximum concentration of 3000 mg/kg/day [18]. Other edible insects *Allomyrina dichotoma* (Coleoptera: *Scrabaeidae*) revealed no adverse effect for 13 weeks (NOAEL: 2500 mg/kg/day) in SD rats [19], and subacute oral toxicity with *Gryllus bimaculatus* (Orthoptera: *Gryllidae*) for 4 weeks in SD rats were performed, and no toxicity were observed (NOAEL: 3000 mg/kg/day) [20]. Recently, *Z. atratus* has been recognized as a temporary food ingredient through the evaluation of suitability and food safety of manufacturing methods by the Korea Ministry of Food and Drug Safety (MFDS) in January 2020.

On the other hand, the risk of potential adverse effects of insect consumption including allergic reactions such as atopic dermatitis and toxic reactions due to food allergies can vary because of differences in geographical food culture [21,22]. For instance, it has been reported that each year in China over 1000 patients experience anaphylactic reactions after ingesting silkworm pupa [23]. In Spain, infestation of lentils with lentil parasite (*Bruchus lentis*) is relatively common and sixteen patients with allergic symptoms related to inhalation or ingestion of lentils containing parasite parts have been evaluated [24]. A deeper study is needed on allergy risk from ingestion of insects and related ingredients, and a great deal of attention is required to discriminate between signs of toxic reactions and allergenicity symptoms [25,26].

Toxicological tests, such as general toxicity or genotoxicity tests, are necessary to ensure safety of human health regarding use of edible insects as a novel food source. The daily maximum intake allowance of edible insects can be calculated based on the results of toxicological tests to provide reliable safety data. To date, adverse effects of *Z. atratus* consumption in humans have not been reported. Therefore, toxicological safety evaluations of long-term exposure should be performed to evaluate the safety of freeze-dried skimmed powdered *Z. atratus* larvae (frpfdZAL) as a novel food source for humans. Thus, the present study of frpfdZAL was designed to evaluate subchronic toxicities including potential allergy reaction of frpfdZAL for human use by performing 2-week and 13-week repeated-dose oral toxicity tests in SD rats following the Good Laboratory Practice (GLP) regulations of the Organization for Economic Cooperation and Development (OECD) [27] and the Korea MFDS [28].

## 2. Materials and Methods

### 2.1. Preparation and Analysis of frpfdZAL

The preparation of frpfdZAL was supported by the National Institute of Agricultural Science (Wanju, Republic of Korea). The larvae were allowed to breed at 26 ± 2 °C, 65% relative humidity, and 12-h/12-h light:dark cycle in a plastic breeding box (27 cm × 36 cm × 8 cm) for 2 days in the Industrial Insect Division, National Institute of Agricultural Science. After the larvae (10th and 16th instar stage) were fasted for 2 days, fecal debris was separated and the remainder was washed twice with flowing water, and then dried. The separated larvae were autoclaved at 115 °C for 5–15 min in a steam sterilizer (Tomy Kogyo, Tokyo, Japan) and then frozen from −70 °C to < 0 °C for more than 24 h in an ultra-low temperature freezer (NIHON freezer, Tokyo, Japan). The larvae were then dried for approximately 65 h using a freeze dryer (NIHON freezer, Tokyo, Japan), and were ground into a powder using a multifunctional crusher (Korea Medi, Daegu, Republic of Korea). Larvae powder containing 21.7% crude fat was prepared by pulverizing using a mesh mill (Garyeo Industry, Siheung, Korea). The resulting pulverized larvae powder was used for the production of skimmed powder. The skimmed powder was grayish brown with the original flavor and odor. The frpfdZAL was examined for food poisoning pathogen contamination by assessing *E. coli* 0157: H7, *Coliform* group, *Salmonella* spp., and *Staphylococcus aureus* and mycotoxin such as Aflatoxin and Ochratoxin, and monitoring testing for heavy metals including lead (Pb), mercury (Hg), arsenic (As), and cadmium (Cd). The test substance was found to be safe from food poisoning pathogens, and heavy metals were undetectable or found at lower than the standard index for food [14] as shown in Table 1. The powder was weighed and suspended in the vehicle, distilled water for injection, by homogenizer and vortexer to reach the target concentration. Dose formulations were prepared on the day of administration. Dose formulation were analyzed the first (Day 1) and the last day (Day 92) of preparation and the recovery rate of free tryptophan concentration was within 100 ± 20% of the target concentration.

### 2.2. Animal and Maintenance

Specific pathogen-free (SPF) SD rats and Hartley guinea pigs were obtained from Samtako Bio Korea Inc (Osan-si, Korea) at 5 weeks of age. The animals were acclimatized for 6 days and healthy animals were selected for the study. Then, 40 male and 40 female rats were assigned randomly to four groups, one vehicle control and three treatment groups, respectively. At the start of dosing, body weight ranged at 180.0 ± 9.07 g for males and 150.92 ± 7.10 g for females. The animals were maintained under constant environmental conditions (temperature, 23 ± 3 °C; humidity, 55 ± 15%; ventilation, 10–20 air changes/h, and luminous intensity, 150–300 Lux) in the barriered experimental animal facility at Chemon Inc. in the Nonclinical Research Institute accredited by the AAALAC International on March 02, 2010 (#001333) in accordance with the Guide for the Care and Use of Laboratory Animals, 8th edition [29]. Food and water were provided, ad libitum, with 12 h light: 12 h dark cycle. All procedures and protocols were reviewed and approved by the Institutional Animal Care and Use Committee (IACUC) of Chemon Inc. performed in accordance with the guideline published by the OECD as well as the GLP regulations for Nonclinical Laboratory Studies of the MFDS in Korea [27,28,30,31].

### 2.3. Experimental Design for Repeated Oral Dose Toxicity Study

For the 13-week repeat-dose toxicity study, in accordance with OECD Guideline 408 [32], healthy male and female 6-week old SD rats were randomly assigned to four groups (10/sex/group) under GLP regulations. Vehicle (distilled water for injection) or graded doses of frpfdZAL (1250, 2500, and 5000 mg/kg of body weight) were administered to rats by oral gavage once daily for 13 weeks at dose of 20 mL/kg of body weight after completion of a 14-day repeated-dose DRF (dose range finding) study, dosing up to 5000 mg/kg/day. The rats were observed daily for clinical signs including mortality, general appearance, and behavioral abnormality until terminal sacrifice. Body weights and food/water consumption were recorded weekly throughout the study. At study termination, all rats were euthanized by isoflurane (2% to 5%) inhalation for blood sample collection.

### 2.4. Urinalysis

In the last week of observation, 5 animals/sex/dose were individually housed in metabolic cages. About 1 mL of fresh urine samples were taken for analysis. The urine was collected for 24 h to obtain the total urine volume for each animal. Urine test strips (Multistix 10SG, Siemens, Munchen, Germany) were dipped in approximately 0.3 mL samples of urine, and the following parameters were analyzed using an automatic analyzer (Clinitek Advantus, Siemens, Munchen, Germany): bilirubin (BIL), pH, nitrite (NIT), ketone body (KET), protein (PRO), occult blood (BLO), glucose (GLU), specific gravity, and urobilinogen (URO). Urine color was observed by naked eye in the animal room, and the results were manually input into the automatic analyzer (COBAS U411 Urine Analyzer; Roche Diagnostics, Mannheim, Germany). For the urine sediment test, the remaining urine sample after general examination was centrifuged at 33× *g* (Combi-514R; Hanil, Gimpo, Korea) for 5 min. The sediments were stained using Sternheimer-Malbin method, and the following parameters were evaluated using a microscope: WBC, epithelial cell, RBC counts, and cast.

### 2.5. Hematology and Clinical Biochemistry

After blood samples were collected into CBC bottles (Vacutainer 3 mL; BD, Franklin Lakes, NJ, USA) containing the anticoagulant EDTA-2K, the following hematological parameters were analyzed using a Coulter counter (Siemens, Tarrytown, NY, USA): red blood cell (RBC) count, platelet (PLT) count, hematocrit (HCT), reticulocyte (RET) count, hemoglobin (HGB) level, white blood cell (WBC) count, mean corpuscular volume (MCV), neutrophil (NEU) count, mean corpuscular hemoglobin level(MCH), lymphocyte (LYM) count, monocyte (MONO) count, mean corpuscular hemoglobin concentration (MCHC), eosinophil (EOS) count, basophil (BASO) count, large unstained cells (LUC), Prothrombin time (PT), and Activated partial thromboplastin time (APTT). The following serum biochemistry parameters were also measured using serum immediately separated from whole blood samples collected into 5-mL Vacutainer tubes (SST™ II Advance, BD, Wokingham, UK) containing clot activator with an automatic chemistry analyzer (AU680; Beckman Coulter, CA, USA): aspartate aminotransferase (AST), alkaline phosphatase (ALP), alanine aminotransferase (ALT), total cholesterol (TCHO), creatinine (CRE), triglyceride (TG), inorganic phosphorus (IP), total protein (TP), calcium ion (Ca^2+^), total bilirubin (TBIL), creatine phosphokinase (CPK), albumin (ALB), sodium ion (Na^+^), albumin/globulin (A/G) ratio, potassium ion (Na^+^), glucose (GLU), blood urea nitrogen (BUN), and chloride ion (Cl^−^).

### 2.6. Gross Findings, Organ Weights, and Histopathological Examinations

At necropsy, the animals were sacrificed to analyze the gross and microscopic features of the internal organs. The absolute weights of brain, spleen, epididymis, pituitary gland, adrenal gland, prostate gland, lung, kidney, ovary, heart, liver, uterus with cervix, thymus, and testis of all rats were weighed using an electronic balance (Secura 224-1S; Sartorius AG, Göttingen, Germany), and the organ to fasted body weight ratios (relative organ weights) were calculated (data not shown). All tissues from each animal were preserved in 10% neutral buffered formalin. The eyes were preserved in Davidson’s solution and the testis and epididymis were preserved in Bouin’s solution. Lesions were graded using a five-step scale in the order of increasing severity (minimal, mild, moderate, severe, and massive). The histopathological findings were processed using Pristima^®^ (Xybion, NJ, USA). The diagnostic terms in Lexicon of Pristima^®^ were used primarily. Standardized System for Nomenclature and Diagnostic Criteria-Guides for Toxicologic Pathology (published by Society of Toxicology (SOT)) and Covance Glossary [published by Covance] were also utilized.

### 2.7. Identification of Allergic Reactions

After sacrifice, approximately 0.6 mL of blood was collected from the cauda vena cava of the rats into tubes (SST™ II Advance). Whole blood was stored at ambient room temperature (22 ± 3 °C) for at least 30 min and centrifuged at 11,700× *g* for 3 min, and the serum was separated. Serum IgE levels were measured using the IgE (Rat) ELISA kit (Abnova Corporation, Taipei City, Taiwan). The results were obtained using the SpectraMax M3 microplate reader (Molecular Devices Corp., Sunnyvale, CA, USA). Data analysis and calculation were conducted using SOFTmax Pro (ver. 5.4.1, Molecular Devices) and Microsoft Office Excel 2010 (Microsoft Corp., Redmond, WA, USA). Data were obtained from a four-parameter logistic curve using fitted calibrations.

### 2.8. Statistical Analysis

Statistical analysis was performed by parametric one-way analysis of variance (ANOVA) followed by multiple comparison with Duncan’s multiple range test (body weights, food and water consumption, urine volume, hematological and clinical biochemistry parameters, organ weights, and IgE), or by non-parametric Kruskal–Wallis H test by the Mann-Whitney U test (the urinalysis data) using SPSS Statistics v22 (Armonk, NY, USA). The level of significance was taken as *p* < 0.05.

## 3. Results

### 3.1. 2-Week Repeated-Dose Toxicity Study

**Figure 1:**
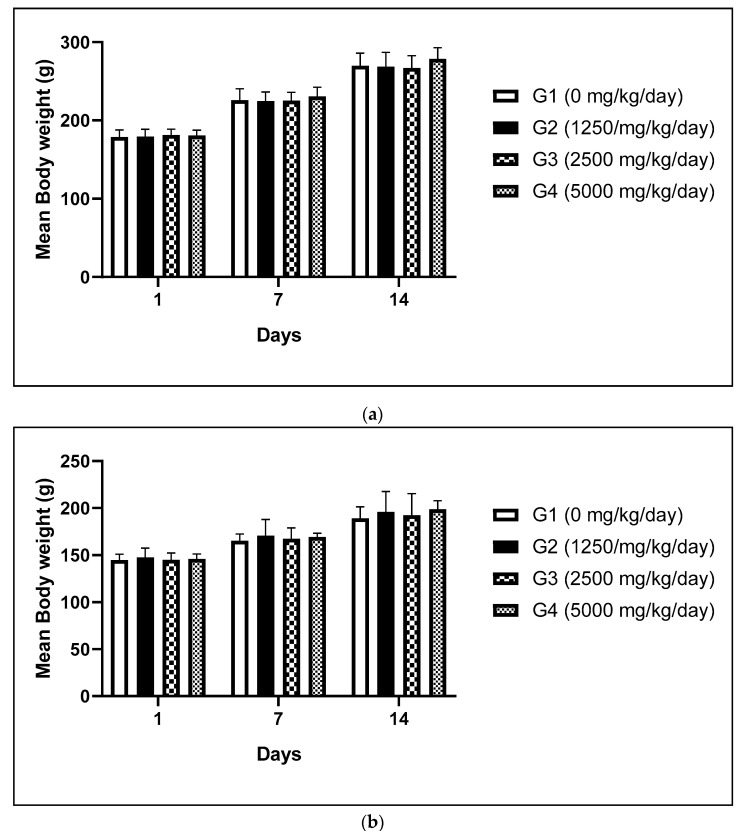
Mean body weight of male (**a**) and female (**b**) rats administered with frpfdZAL for 2 weeks. Each value represents the mean ± standard deviation (*n* = 5). (**a**) male; (**b**) female.

There is still insufficient toxicological information on the oral toxicity of frpfdZAL after long-term exposures. Therefore, repeated-dose toxicity study of frpfdZAL at doses of 1250, 2500 and 5000 mg/kg/day administered by oral gavage for 14 days was carried out to assess toxicity. As a result, no test substance-related changes in mortalities, clinical signs, body weights, food and water consumption, ophthalmological examination, urinalysis, hematological and clinical biochemistry tests, organ weight, and gross findings were observed during 2-week treatment period (body weights as shown in Figure 1.

### 3.2. 13-Week Repeated-Dose Toxicity Study

Accordingly, the results of the 2-week repeated-dose oral toxicity study were used to provide a rational for selection of dose levels. Therefore, 5000 mg/kg/day was selected as the high dose, and an additional 2 groups were set as the middle- and low-dose using 2-fold intervals. When rats were given a 13-week repeated-dose oral administration of frpfdZAL at doses of 1250, 2500, and 5000 mg/kg/day, assessment of toxicity was based on clinical signs, body weights, food and water consumption, ophthalmological examinations, urinalysis, hematological test, clinical biochemistry test, organ weights, gross findings, and histopathological examinations.

#### 3.2.1. Clinical Observation, Body Weight, Food and Water Consumption, and Ophthalmologic Examination

**Figure 2:**
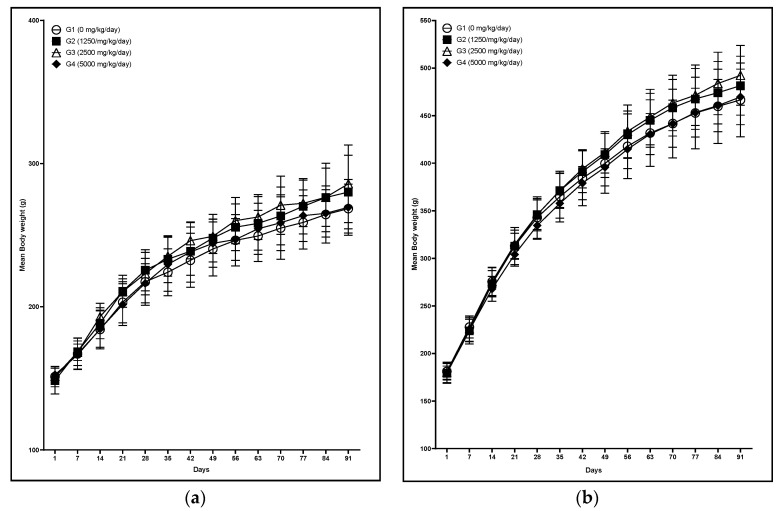
Mean body weight of male (**a**) and female (**b**) rats administered with frpfdZAL for 13 weeks. Each value represents the mean ± standard deviation (*n* = 10). (**a**) male; (**b**) female.

In clinical observation, reddish tear was observed in one male and loss of fur was observed in one male and one female was observed at 5000 mg/kg/day. No test substance-related changes were observed in body weights, food and water consumption between vehicle control and test substance-treated groups. No abnormalities were found in either sex of any group at ophthalmologic examination. Body weight changes are presented in Figure 2. In addition, frpfdZAL did not affect body weight gains at any dose throughout the study period (data not shown).

#### 3.2.2. Urinalysis, Hematological Tests and Clinical Biochemistry Tests

KET significantly increased in males at 5000 mg/kg/day (*p* < 0.05) and pH significantly decreased in females at 5000 mg/kg/day (*p* < 0.05) as shown in Table 2. However, no test substance-related changes in microscopic examination for sediments of WBC, epithelial cell, and RBC counts, and cast were observed (data not shown). In the hematological tests, statistically significant change in WBC noted in males was observed (*p* < 0.01). Also, ALP significantly increased in females at 2500 mg/kg/day (*p* < 0.05), and TP and ALB significantly decreased in females at 5000 mg/kg/day (*p* < 0.01) in the clinical biochemistry tests. The hematological and clinical biochemistry values are presented in Table 3 and Table 4, respectively.

#### 3.2.3. Gross Findings, Organ Weights, and Histopathological Examinations

No test substance-related changes were observed in the weight of any organ. In macroscopic or histopathological examinations, no test substance-related toxicological lesions were observed in any group (data not shown). The absolute organ weights are presented in Table 5.

#### 3.2.4. Allergic Reaction

There were no significant changes in IgE concentrations in males of any group throughout the study period. As shown in Table 6, the IgE concentrations in this study were close to basal level of total IgE in rats and there was no dose-dependent relationship. Therefore, frpfdZAL did not induce increases of serum IgE, an identifier of allergic reactions in rats.

## 4. Discussion

Insects, a number of new sources for food and feed proteins, have been consumed by humans for thousands of years due to their proported nutritional and pharmacological benefits [33]. However, insect proteins have to be checked for food and feed safety. In this respect, potential toxicity and adequate food safety of insects are frequently debated. Therefore, toxicological assays were performed to evaluate the safety of frpfdZAL as an edible insect in 2-week and 13-week repeated-dose oral toxicity studies in compliance with OECD guidelines under GLP regulations.

As a result, frpfdZAL did not induce acute toxicity in rats and the approximate lethal dose was greater than 5000 mg/kg (data not shown). In addition, no toxicological changes were observed in the 2-week and 13-week repeated-dose oral toxicity studies. In clinical signs, reddish tear and loss of fur observed were considered toxicologically insignificant because reddish tear was observed in only one animal throughout the study period, and loss of fur observed in only one animal has often been found in SD rat studies. Table 1 showing the nutritional composition analysis of frpfdZAL, the content of crude protein (61.39%) is elatively high when compared to other contents such as fat (21.24%), fiber (7.04%), and ash (4.54%). Therefore, one consideration is that such high protein intake could affect kidney and liver function or cause impairment [34,35]. However, Clifton et al. [36] reported that high protein intake has been associated with body weight loss and positive metabolic effects in obese people. On the contrary, it has been reported [37] that the effects of long-term intake of high protein diets are related to a higher risk of weight gain and associated with increased risk of fatal and non-fatal outcomes. It has also been reported that long-term intake of a high protein diet results in TG deposition, increased inflammation, alterations in the acid-base equilibrium, and oxidative stress [38]. In kidney, high protein intake leads to increased glomerular filtration rate (GFR), causing ‘glomerular hyperfiltration’ as a result of the amino acid surge, which leads to dilatation of the afferent arteriole, and increases intraglomerular pressure [39]. On the contrary, a lower intake of dietary protein brings out more constriction of the afferent arteriole, resulting in decreased intraglomerular pressure and lowered GFR [40]. Interestingly, chronic progressive nephropathy was found in 100% of control male rats in the 90-day toxicity studies, regardless of NIH-07 or NTP-2000 diets or route of exposure [41]. However, under present laboratory conditions of the study, total protein concentration and liver-related parameters in the clinical biochemistry test were considered to be of little toxicological significance and were within the normal physiological ranges [25,42], even in combination with the high protein level (61.39%) frpfdZAL and normal rodent feed containing more than 18% of crude protein every day. However, high protein levels in frpfdZAL could be attributed to significant changes of KET in males or pH in females at 5000 mg/kg/day in urinalysis. However, these changes were not considered to be toxicologically significant since the related organ weights and histopathological findings in organs such as kidney and liver were not significantly changed. Thus, even if the biochemical indicators and histopathology of kidney and liver are considered irrelevant in SD rats in these oral toxicity studies, care should be taken in patients with kidney or liver failure when ingesting high-protein frpfdZAL as a dietary additive.

Food allergy is recognized as an adverse effect arising from immune response after exposure to a given food [43,44,45]. As part of this concern, edible insects need to be characterized with particular focus given to nutritional value as well as other safety-related factors such as existence of microorganisms, toxins, and heavy metals [46]. Since there are no detailed regulations on how to assess the risk of allergies in new foods, various methods were proposed to assess the allergic potential of new food protein and protein sources [45]. Specifically, it has been reported [47] that some allergic components in insect-derived proteins are recognized to be arginine kinase, tropomyosin, light and heavy chain myosin, and larval cuticle proteins, resulting in sensitization and insect cross-reactivity. Regarding sensitizations to insect-derived proteins, total serum IgE is defined as an indication of allergic components [48]. Therefore, we conducted allergic reaction test to check IgE concentration in rats given 13-week repeated-dose administration of frpfdZAL. As shown in Table 6, no significant change of IgE levels was observed in the four groups throughout the study period. The serum IgE level after treatment with frpfdZAL was similar to that of the vehicle control (at 14.091 ± 1.725 ng/mL). In addition, Han et al., [18] reported that identifications of allergic reaction such as IgE or histamine concentration in blood were not significant tests of yellow mealworm extracts. Interestingly, the level of total IgE in SD rats treated with frpfdZAL was similar to the yellow mealworm-treated SD rat blood samples [18]. In addition, allergenicity-related WBC parameter such as BASO was not significant or similar to the vehicle control groups in hematological tests as shown in Table 3. However, allergic reaction testing, in itself, was not enough to determind all adverse immune responses in this study. Therefore, further evaluative toxicity studies involving in allergenicity should be performed to investigate immune responses related with cytokine release or mast cells activation after insect protein ingestion.

Since 2013, *Z. atratus* was recognized and consumed as a food ingredient in Belgium, Netherlands and Australia [49,50,51]. Around the same time, the South Korea’s edible insect food market has grown greatly thanks to government support and intensive research efforts. Recently, as part of food allergy, a set of multiplex polymerase chain reaction (mPCR) has been developed to detect edible insects directly in dietaries to inform consumers who are allergic to certain edible insect in advance of what kinds of edible insects are contained in the foods using labeling of products [52]. In addition to the nutritional values as food, Han et al. [53] reported that identifying the physiologically crucial ingredients of edible insects is a very significant process that allows to be potentially used for medical applications. In spite of the great advantages in the edible insects, the food safety issues of microorganism contamination, allergenicity, and toxicity should be carefully evaluated to confirm the considerable nutritional and medicinal values contained in insect food sources. The efficacy and suitability for human and animal consumption of insects as health food or medicinal ingredients, or use in animal feed must be explored further. Therefore, frpfdZAL is one insect industry candidate which has the potential for utilization as a suitable ingredient in both animal feed and human food.

## 5. Conclusions

Based on the above results, when rats were given 2-week or 13-week repeated-dose oral administration of frpfdZAL, at up to 5000 mg/kg/day, the NOAEL was considered to be 5000 mg/kg/day and no target organ was identified in both sexes under the experimental conditions of this study. In addition, frpfdZAL did not induce increases of IgE, an identifier of allergic reactions in rats. Collectively, these results suggest that frpfdZAL is safe with no adverse effects up to 5000 mg/kg/day and, therefore, may be suitable for use as an edible ingredient in both animal feeds and human foods at levels below 5000 mg/kg/day.

## Figures and Tables

**Table 1:** The nutritional components of frpfdZAL.

General Components	Compositional Average (%)
Moisture	2.34
Crude protein	61.39
Crude fat	21.24
Total carbohydrate	3.45
Crude ash	4.54
Crude fiber	7.04
**Vitamin**	**Contents (mg/100g)**
Vitamin A	N.D. ^†^
Vitamin D	N.D. ^†^
Vitamin E (Tocopherol)	1.20
Vitamin B3 (Niacin)	203.24
Vitamin B5 (Pantothenic acid)	N.D. ^†^
Vitamin B6 (Pyridoxine)	N.D. ^†^
Vitamin B12 (Cobalamin)	N.D. ^†^
Vitamin C	5.78
**Mineral**	**Contents (mg/100g or µg/100g)**
Calcium (Ca)	40.17
Phosphorus (P)	1029.45
Potassium (K)	1267.04
Magnesium (Mg)	257.28
Iron (Fe)	5.28
Zinc (Zn)	12.33
Copper (Cu)	1.22
Manganese (Mn)	1.45
Selenium (Se)	65.73 ug/100g
**Mycotoxin**	**Contents (µg/kg)**
Total Aflatoxins (Aflatoxin B1, B2, G1, G2)	N.D. ^†^
Ochratoxin A	N.D. ^†^
**Food Poisoning Bacteria**	**Qualitative Analysis**
*E. coli* 0157:H7	Negative
Coliform group	Negative
*Salmonella* spp.	Negative
*Staphylococcus aureus*	Negative
**Heavy Metal**	**Contents (mg/kg)**
Lead (Pb)	0.03
Cadmium (Cd)	0.08
Arsenic (As)	0.02
Mercury (Hg)	N.D. ^†^

^†^ N.D., Not Detected.

**Table 2:** Urinalysis data of SD rats orally administered frpfdZAL for 13 weeks.

Parameters	Result	0 mg/kg/day	1250 mg/kg/day	2500 mg/kg/day	5000 mg/kg/day
Male					
GLU	-	5	5	5	5
BIL	-	5	5	5	5
KET	-	5	5	5	1
	+/−	0	0	0	3
	1+	0	0	0	1 *
pH	7.0	0	0	0	0
	7.5	1	1	2	0
	8.0	3	1	3	1
	8.5	1	3	0	4
PRO	-	0	2	1	1
	+/−	3	3	2	0
	1+	2	0	2	4
URO	0.2	5	5	5	5
NIT	-	5	5	5	5
BLO	-	1	3	4	3
	+/−	4	2	1	2
volume	(mL)	15.8 ± 3.6	14.6 ± 2.1	15.4 ± 2.2	14.2 ± 3.3
Female					
GLU	-	5	5	5	5
BIL	-	5	5	5	5
KET	-	5	5	5	5
pH	7.0	0	0	0	2 *
	7.5	0	0	0	2
	8.0	1	1	4	0
	8.5	4	4	1	1
PRO	+/−	5	5	5	4
	1+	0	0	0	1
URO	0.2	5	5	5	5
NIT	-	5	5	4	4
	+	0	0	1	1
BLO	+/−	4	2	1	2
volume	(mL)	11.2 ± 2.9	11.0 ± 4.5	9.8 ± 7.7	11.2 ± 3.1

GLU: Glucose, BIL: Bilirubin, KET: Ketone body, PRO: Protein, URO: Urobilinogen, NIT: Nitrite, BLO: Occult blood. * Significant difference at *p* < 0.05 levels compared to the vehicle control (*n* = 5). Citation in Table A1 for Urinalysis data.

**Table 3:** Hematological data of SD rats orally administered frpfdZAL for 13 weeks.

Parameters	0 mg/kg/day	1250 mg/kg/day	2500 mg/kg/day	5000 mg/kg/day
Males				
RBC (10^6^/μL)	9.03 ± 0.27	8.89 ± 0.38	8.91 ± 0.25	8.93 ± 0.33
HGB (g/dL)	15.4 ± 0.4	15.0 ± 0.4	15.2 ± 0.3	15.4 ± 0.3
HCT (%)	47.7 ± 1.2	46.7 ± 1.0	47.5 ± 0.9	47.8 ± 1.3
MCV (fL)	52.9 ± 1.3	52.7 ± 2.3	53.4 ± 0.9	53.6 ± 0.9
MCH (pg)	17.0 ± 0.5	16.9 ± 0.9	17.1 ± 0.3	17.2 ± 0.4
MCHC (g/dL)	32.2 ± 0.3	32.0 ± 0.5	32.0 ± 0.3	32.2 ± 0.5
RET (%)	2.06 ± 0.31	2.12 ± 0.31	2.20 ± 0.24	2.05 ± 0.26
PLT (10^3^/μL)	899.5 ± 57.4	885.7 ± 71.7	866.9 ± 119.7	853.6 ± 54.8
WBC (10^3^/μL)	7.14 ± 1.10	6.53 ± 1.42	6.28 ± 1.13	5.53 ± 0.85 **
NEU (%)	22.8 ± 8.0	15.1 ± 5.1	21.1 ± 6.9	19.3 ± 4.4
LYM (%)	72.3 ± 8.1	79.4 ± 5.3	73.4 ± 6.9	74.8 ± 5.4
MONO (%)	2.51 ± 0.48	3.09 ± 1.13	3.02 ± 0.86	3.74 ± 1.57
EOS (%)	1.77 ± 0.54	1.68 ± 0.39	1.75 ± 0.55	1.51 ± 0.32
BASO (%)	0.15 ± 0.07	0.16 ± 0.07	0.18 ± 0.06	0.14 ± 0.05
LUC (%)	0.49 ± 0.16	0.59 ± 0.23	0.56 ± 0.26	0.52 ± 0.23
PT (sec)	8.5 ± 0.3	8.5 ± 0.3	8.6 ± 0.1	8.7 ± 0.3
APTT (sec)	13.9 ± 1.4	13.8 ± 1.7	14.2 ± 1.1	15.3 ± 1.7
Females				
RBC (10^6^/μL)	7.86 ± 0.53	7.81 ± 0.40	7.78 ± 0.28	7.98 ± 0.58
HGB (g/dL)	14.1 ± 0.5	14.4 ± 0.5	14.2 ± 0.3	14.4 ± 1.0
HCT (%)	43.4 ± 1.7	44.4 ± 1.8	43.6 ± 1.2	44.2 ± 3.1
MCV (fL)	55.3 ± 1.9	56.9 ± 1.6	56.1 ± 1.0	55.5 ± 1.1
MCH (pg)	18.0 ± 0.7	18.5 ± 0.5	18.3 ± 0.5	18.0 ± 0.4
MCHC (g/dL)	32.5 ± 0.5	32.6 ± 0.3	32.5 ± 0.5	32.5 ± 0.4
RET (%)	2.51 ± 0.45	2.41 ± 0.44	2.60 ± 0.39	2.30 ± 0.31
PLT (10^3^/μL)	956.9 ± 76.5	857.4 ± 66.7	929.0 ± 114.8	966.5 ± 107.2
WBC (10^3^/μL)	3.89 ± 1.12	3.29 ± 0.94	3.13 ± 0.85	3.30 ± 0.88
NEU (%)	13.6 ± 3.2	13.5 ± 5.2	15.1 ± 6.8	16.1 ± 18.4
LYM (%)	81.0 ± 3.1	81.6 ± 6.1	78.9 ± 6.7	78.3 ± 19.7
MONO (%)	2.88 ± 0.93	2.40 ± 0.79	2.93 ± 0.92	2.91 ± 1.58
EOS (%)	1.76 ± 0.64	1.80 ± 0.47	2.39 ± 0.84	2.12 ± 1.02
BASO (%)	0.12 ± 0.08	0.13 ± 0.05	0.10 ± 0.07	0.10 ± 0.07
LUC (%)	0.63 ± 0.41	0.62 ± 0.21	0.64 ± 0.41	0.46 ± 0.16
PT (sec)	8.0 ± 0.3	8.2 ± 0.2	8.1 ± 0.2	8.1 ± 0.2
APTT (sec)	12.8 ± 1.6	13.2 ± 1.3	12.8 ± 1.5	13.2 ± 0.8

Each value represents the mean ± standard deviation (*n* = 10). WBC, white blood cell; RBC, red blood cell; HGB, hemoglobin; HCT, hematocrit; MCV, mean corpuscular volume; MCH, mean corpuscular hemoglobin; MCHC, mean corpuscular hemoglobin concentration; PLT, platelet; RET, reticulocyte; PT, prothrombin time; APTT, activated partial thromboplastin time; NEU, Neutrophil; LYM, Lymphocyte; MONO, Monocyte; EOS, Eosinophil; BASO, Basophil; LUC, Large unstained cells.

**Table 4:** Clinical Biochemistry data of SD rats orally administered frpfdZAL for 13 weeks.

Parameters	0 mg/kg/day	1250 mg/kg/day	2500 mg/kg/day	5000 mg/kg/day
Males				
AST (U/L)	106.1 ± 37.7	118.5 ± 43.3	111.2 ± 23.4	106.4 ± 21.1
ALT (U/L)	42.8 ± 11.8	42.3 ± 16.0	40.5 ± 10.6	42.2 ± 10.3
ALP (U/L)	89.6 ± 20.1	92.0 ± 26.5	87.5 ± 19.2	81.4 ± 12.4
CPK (U/L)	381.9 ± 187.6	353.8 ± 156.8	392.3 ± 298.0	412.8 ± 149.7
TBIL (mg/dL)	0.13 ± 0.02	0.15 ± 0.03	0.14 ± 0.03	0.14 ± 0.03
GLU (mg/dL)	163.6 ± 29.0	158.5 ± 12.9	155.0 ± 17.9	167.4 ± 19.1
TCHO (mg/dL)	86.9 ± 10.0	81.0 ± 12.7	82.1 ± 14.4	74.1 ± 12.7
TG (mg/dL)	57.8 ± 14.9	63.5 ± 26.1	64.3 ± 26.1	55.9 ± 12.1
TP (g/dL)	6.27 ± 0.19	6.29 ± 0.23	6.24 ± 0.21	6.29 ± 0.22
ALB (g/dL)	2.88 ± 0.06	2.89 ± 0.05	2.86 ± 0.07	2.86 ± 0.10
A/G ratio	0.85 ± 0.03	0.85 ± 0.04	0.85 ± 0.04	0.84 ± 0.03
BUN (mg/dL)	15.3 ± 1.4	14.8 ± 1.0	14.8 ± 1.6	15.1 ± 1.1
CRE (mg/dL)	0.41 ± 0.03	0.44 ± 0.02	0.42 ± 0.02	0.41 ± 0.02
Females				
AST (U/L)	90.3 ± 20.5	94.7 ± 34.6	88.4 ± 21.0	106.2 ± 21.7
ALT (U/L)	29.5 ± 5.7	36.9 ± 14.3	31.6 ± 3.6	34.6 ± 6.7
ALP (U/L)	53.4 ± 14.3	51.9 ± 14.3	67.6 ± 11.8 *	62.1 ± 12.5
CPK (U/L)	239.3 ± 109.6	275.3 ± 226.9	250.2 ± 165.1	294.3 ± 206.8
TBIL (mg/dL)	0.16 ± 0.04	0.17 ± 0.03	0.16 ± 0.02	0.17 ± 0.05
GLU (mg/dL)	129.9 ± 16.0	113.5 ± 9.8	122.5 ± 14.8	134.1 ± 31.9
TCHO (mg/dL)	96.3 ± 12.2	90.3 ± 22.4	94.9 ± 9.5	92.2 ± 18.7
TG (mg/dL)	41.5 ± 9.0	39.9 ± 6.9	39.5 ± 6.6	37.1 ± 8.6
TP (g/dL)	6.34 ± 0.24	6.19 ± 0.18	6.26 ± 0.16	6.01 ± 0.28 **
ALB (g/dL)	3.18 ± 0.11	3.12 ± 0.13	3.16 ± 0.16	2.99 ± 0.11 **
A/G ratio	1.01 ± 0.04	1.02 ± 0.05	1.02 ± 0.06	0.99 ± 0.06
BUN (mg/dL)	18.6 ± 3.3	16.5 ± 1.5	17.6 ± 0.9	17.4 ± 1.3
CRE (mg/dL)	0.52 ± 0.06	0.50 ± 0.04	0.51 ± 0.02	0.50 ± 0.05

Each value represents the mean ± standard deviation (*n* = 10). GLU, glucose; BUN, blood urea nitrogen; CREA, creatinine; TP, total protein; ALB, albumin; A/G, albumin/globulin ratio; AST, aspartate aminotransferase; ALT, aminotransferase; ALP, Alkaline phosphatase; CK, creatine kinase; TCHO, total cholesterol; TG, triglyceride; TBIL, Total bilirubin. */** Significant difference at *p* < 0.05/*p* < 0.01 levels compared to the vehicle control.

**Table 5:** Organ weights of SD rats orally administered frpfdZAL for 13 weeks.

Parameters	Organ Weight (g)			
	0 mg/kg/day	1250 mg/kg/day	2500 mg/kg/day	5000 mg/kg/day
Males				
Body weights	440.27 ± 37.43	457.95 ± 29.97	469.15 ± 30.83	445.98 ± 26.49
Pituitary gland	0.014 ± 0.003	0.01 ± 0.01	0.01 ± 0.01	0.013 ± 0.01
Prostate gland	0.80 ± 0.18	0.75 ± 0.17	0.75 ± 0.17	0.79 ± 0.25
Testis	3.87 ± 0.32	3.86 ± 0.36	3.91 ± 0.47	3.67 ± 0.24
Epididymis	1.39 ± 0.13	1.40 ± 0.16	1.41 ± 0.12	1.38 ± 0.09
Brain	2.13 ± 0.10	2.11 ± 0.06	2.13 ± 0.09	2.16 ± 0.13
Liver	11.80 ± 1.11	11.77 ± 0.89	12.97 ± 1.65	12.02 ± 0.85
Kidney	2.72 ± 0.27	2.63 ± 0.22	2.85 ± 0.21	2.82 ± 0.35
Thymus	0.29 ± 0.05	0.31 ± 0.08	0.28 ± 0.07	0.25 ± 0.05
Adrenal gland	0.05 ± 0.01	0.05 ± 0.01	0.05 ± 0.01	0.05 ± 0.01
spleen	0.76 ± 0.080	0.86 ± 0.18	0.84 ± 0.14	0.80 ± 0.07
Lung	1.59 ± 0.13	1.65 ± 0.14	1.66 ± 0.16	1.59 ± 0.12
Heart	1.31 ± 0.11	1.35 ± 0.12	1.36 ± 0.11	1.28 ± 0.09
Females				
Body weights	252.52 ± 15.74	263.53 ± 23.05	270.42 ± 25.17	253.26 ± 19.81
Ovary	0.08 ± 0.015	0.091 ± 0.02	0.10 ± 0.02	0.09 ± 0.010
Pituitary gland	0.02 ± 0.006	0.016 ± 0.01	0.02 ± 0.01	0.02 ± 0.01
Uterus (with cervix)	0.60 ± 0.13	0.84 ± 0.48	0.63 ± 0.34	0.68 ± 0.21
Brain	1.97 ± 0.06	2.01 ± 0.10	1.95 ± 0.08	1.92 ± 0.09
Liver	7.00 ± 0.52	6.74 ± 0.66	7.03 ± 0.78	6.63 ± 0.64
Kidney	1.61 ± 0.15	1.69 ± 0.17	1.69 ± 0.14	1.58 ± 0.19
Thymus	0.25 ± 0.04	0.26 ± 0.05	0.26 ± 0.06	0.23 ± 0.05
Adrenal gland	0.06 ± 0.01	0.06 ± 0.01	0.07 ± 0.01	0.07 ± 0.01
spleen	0.61 ± 0.07	0.62 ± 0.09	0.61 ± 0.08	0.55 ± 0.09
Lung	1.27 ± 0.10	1.29 ± 0.08	1.29 ± 0.14	1.26 ± 0.21
Heart	0.87 ± 0.08	0.89 ± 0.08	0.90 ± 0.08	0.91 ± 0.11

Each value represents the mean ± standard deviation (*n* = 10).

**Table 6:** Serum IgE level of SD rats orally administered frpfdZAL for 13 weeks.

Groups	Serum IgE Levels (ng/mL)
0 mg/kg/day	14.09 ± 1.73
1250 mg/kg/day	14.44 ± 1.32
2500 mg/kg/day	14.36 ± 1.14
5000 mg/kg/day	12.62 ± 0.94

Each value represents the mean ± standard deviation (*n* = 10).

## Appendix A

**Table A1:** For urinalysis data.

RESULT	GLU (mg/dL)	BIL	KET (mg/dL)	PRO (mg/dL)	URO (EU/dL)	NIT	BLO
-	Negative	Negative	Negative	Negative	0.2	Negative	Negative
+/−	100	NA	Trace	15	1.0	NA	Trace
+	NA	NA	NA	NA	NA	Positive	NA
1+	250	Small	15	30	2.0	NA	Small
2+	500	Moderate	40	100	4.0	NA	Moderate
3+	≥1000	Large	≥80	≥300	≥8.0	NA	Large

-, negative; NA, Not applicable.
